# In-hospital mortality among hospitalized COVID-19 patients in a tertiary care hospital in Dhaka City: a retrospective cohort study

**DOI:** 10.3389/fmed.2025.1571865

**Published:** 2025-05-20

**Authors:** Ahmed Hossain, Md. Maruf Hasan, S. M. Sorowar Kamal, Shahnewaz Dewan, Gias U. Ahsan, Juwel Rana

**Affiliations:** ^1^College of Health Sciences, University of Sharjah, Sharjah, United Arab Emirates; ^2^Research and Innovation, South Asian Institute for Social Transformation (SAIST), Dhaka, Bangladesh; ^3^Faculty of Medical Studies, Bangladesh University of Professionals, Dhaka, Bangladesh; ^4^Radiology and Imaging, Sheikh Russel National Gastroliver Institute & Hospital, Dhaka, Bangladesh; ^5^Department of Nephrology, Kurmitola General Hospital, Dhaka, Bangladesh; ^6^Department of Public Health, Daffodil International University, Dhaka, Bangladesh; ^7^Department of Epidemiology, Biostatistics and Occupational Health, Faculty of Medicine and Health Sciences, McGill University, Montreal, QC, Canada

**Keywords:** COVID-19, mortality, risk factors, hospitalized patients, city, Bangladesh

## Abstract

**Background:**

In-hospital mortality during COVID-19 treatment is a crucial metric used to assess the severity of the disease and the effectiveness of medical interventions. By identifying mortality risk factors, we aim to inform policy decisions, optimize resource allocation, and improve preparedness for future pandemics.

**Methods:**

This retrospective cohort study was conducted at a tertiary hospital in Dhaka City, Bangladesh. Data were gathered from the hospital’s electronic medical records between July 2021 and September 2021. After applying specific inclusion and exclusion criteria, 218 patients with complete medical records were selected for the analysis. The independent variables examined included demographic characteristics, comorbidities, and clinical features. To assess in-hospital mortality, relative risks (RR) and 95% confidence intervals were calculated using multivariable logistic regression analysis employing the Delta method.

**Results:**

The study included 218 hospitalized COVID-19 patients, primarily male (51.4%) with an average age of 56.4 years (standard deviation of 15 years). The overall in-hospital mortality rate was 18.3%. Older age (≥60) (RR:3.10, 95% CI: 1.16–8.29), long-standing hypertension (≥5 years) (RR:2.78, 95% CI:1.54–5.02), and chronic kidney disease (CKD) (RR:4.43, 95% CI:2.93–6.70) were significant risk factors for mortality. Patients with diabetes (≥3 years) had a moderately increased risk (RR:1.68, 95% CI: 1.01–2.83). Notably, shorter hospital stays (≤7 days) were associated with higher mortality, potentially due to delayed treatment initiation. Moreover, Vaccinated patients have a significantly lower risk of death (RR: 0.07) compared to unvaccinated patients, highlighting the protective effect of vaccination. Greater lung involvement (especially in lower lobes) and higher Total Severity Scores (TSS ≥ 14) strongly predict COVID-19 mortality, with non-survivors exhibiting significantly worse radiographic damage.

**Conclusion:**

Age, particularly when combined with chronic conditions like hypertension or chronic kidney disease, is a key predictor of in-hospital COVID-19 mortality. While gender is not an independent risk factor, males tend to have higher mortality rates. Delayed treatment, reflected by shorter hospital stays, also increases risk. Vaccination markedly lowers mortality. In resource-limited settings, lower lobe involvement >50% and TSS ≥ 14 can serve as early triage markers to guide ICU admission or intensified care. These indicators should inform risk assessment tools, resource allocation, and targeted interventions to reduce pandemic-related mortality.

## 1 Introduction

The COVID-19 pandemic has placed an unprecedented strain on healthcare systems worldwide, leading to significant morbidity and mortality, especially in hospitalized patients (1). Understanding the factors associated with in-hospital mortality among COVID-19 patients has become a crucial area of research, particularly in tertiary care hospitals where critically ill patients are treated. This study aims to investigate the in-hospital mortality of COVID-19 patients, focusing on the impact of demographic factors, comorbidities, and clinical management strategies.

Several studies have documented mortality trends in COVID-19 patients, highlighting considerable variation across regions, healthcare settings, and populations (2–5). Globally, in-hospital mortality rates have ranged widely, with some studies reporting rates as low as 5% in mild cases, while others have documented rates exceeding 30% in severely affected populations (4–7). Research from high-income countries like the United States and European nations shows that mortality is largely driven by patient age, the presence of underlying health conditions, and access to intensive care, including mechanical ventilation and oxygen therapy (4–7).

In South Asia, including Bangladesh, the healthcare system is less equipped to handle large numbers of critical cases compared to high-income countries (8, 9). Studies from India, Pakistan, and Bangladesh indicate that in-hospital mortality rates in these regions are influenced by factors such as healthcare resource limitations, late presentation to hospitals, and inadequate access to advanced treatments (8–10). A study from a tertiary care hospital in India reported an in-hospital mortality rate of 28.9% among COVID-19 patients, significantly influenced by comorbidities such as diabetes and cardiovascular disease (11).

One of the most consistent predictors of in-hospital mortality in COVID-19 patients is the presence of comorbid conditions such as hypertension, diabetes, cardiovascular diseases, and chronic respiratory conditions (12, 13). Studies worldwide have demonstrated that patients with multiple comorbidities have a higher risk of mortality due to COVID-19. A meta-analysis involving over 50,000 COVID-19 patients found that diabetes, hypertension, and chronic obstructive pulmonary disease (COPD) were associated with significantly higher odds of death (14). Numerous studies and real-world data have demonstrated that COVID-19 vaccines effectively prevent severe illness, hospitalization, and death (15–17).

Age is one of the most significant predictors of COVID-19 mortality, with older patients exhibiting much higher mortality rates than younger cohorts (18). In many studies, the risk of death increases sharply for patients aged 60 and above (18–20). A retrospective study from Wuhan, China, found that patients aged 65 years or older had a mortality rate of 32.8% compared to 1.4% in those under 65.

The severity and mortality of COVID-19 are shaped by a range of factors, including host characteristics (such as age and comorbidities) and viral factors (such as specific SARS-CoV-2 variants). Different variants exhibit distinct profiles in terms of transmissibility, immune evasion, and virulence. For example, the Alpha (B.1.1.7) and Delta (B.1.617.2) variants were linked to increased disease severity and higher mortality compared to the original strain. In contrast, the Omicron variant (B.1.1.529) was generally associated with milder illness but demonstrated greater immune escape.

Tertiary care hospitals are the cornerstone for treating severe cases of COVID-19, especially in resource-constrained settings like Dhaka. Several factors may contribute to in-hospital mortality among COVID-19 patients. These may include limited ICU capacity, inadequate ventilation support, and a high patient-to-doctor ratio. A retrospective study from a tertiary hospital in Iran reported that late presentation, particularly among patients with severe respiratory distress, significantly increased mortality risk (21). Furthermore, socioeconomic factors, such as low health literacy and delayed access to care, may exacerbate mortality rates in this population.

In Bangladesh, particularly in Dhaka City, the high population density and limited healthcare infrastructure have exacerbated the impact of the pandemic. Tertiary hospitals in Dhaka, serving as referral centers for critically ill patients, have seen high mortality rates among COVID-19 patients. This study aims to identify the potential risk factors associated with mortality in hospitalized COVID-19 patients in a tertiary hospital in Dhaka, thereby contributing to more effective management strategies.

## 2 Materials and methods

### 2.1 Study design and setting

This retrospective cohort study was conducted at Kurmitola General Hospital in Dhaka City, Bangladesh. This hospital played a crucial role in the COVID-19 response, starting with patient care in May 2020 and later contributing to vaccination efforts. The hospital is one of the largest in the region and has been at the forefront of treating COVID-19 patients since the beginning of the pandemic. This tertiary hospital, like other hospitals in Dhaka, faced challenges related to resource allocation, particularly during peaks in COVID-19 cases. This hospital, over time, followed a standardized treatment protocol, contributing to better outcomes for some patients, although disparities in care persist due to the resource constraints of the healthcare system. Although including vaccine dosage parameters (e.g., single vs. double dose regimens or booster status) might have enhanced the precision of our COVID-19 mortality analysis, these comparisons were precluded by inconsistent dosage documentation across vaccine types in our dataset. However, it should be noted that all vaccinated patients in our cohort received the AstraZeneca vaccine, as this was the sole formulation available through Bangladesh’s national immunization program during the study period. Consequently, inter-vaccine dosage comparisons were not feasible, and this variable was excluded from our analytical models.

During the outbreak of the delta variant in 2021 in Bangladesh, people sought treatment from hospitals, and case severity was higher than anticipated. Bangladesh faced the third wave of SARS-CoV-2 from May to September 2021, confirming the transmission of the delta variant. To avoid unnecessary hospital admissions, only COVID-19-positive patients with complications (e.g., respiratory distress with SPO_2_ ≤ 89%, co-morbidities, or any other symptoms) were admitted to the hospital for treatment.

### 2.2 Data collection

Data were collected from the hospital’s electronic medical records between July 2021 and September 2021. The data was accessed on October 3, 2023. The study included patients aged 18 years and above who tested positive for COVID-19 by a laboratory test of RT PCR and were admitted to the hospital. The dataset was constrained by RT-PCR sequencing, available for patients with confirmed Delta variant (B.1.617.2) infections. As a result, comprehensive variant-based comparisons were not possible, and viral variant data were excluded from our analysis.

**Figure 1:**
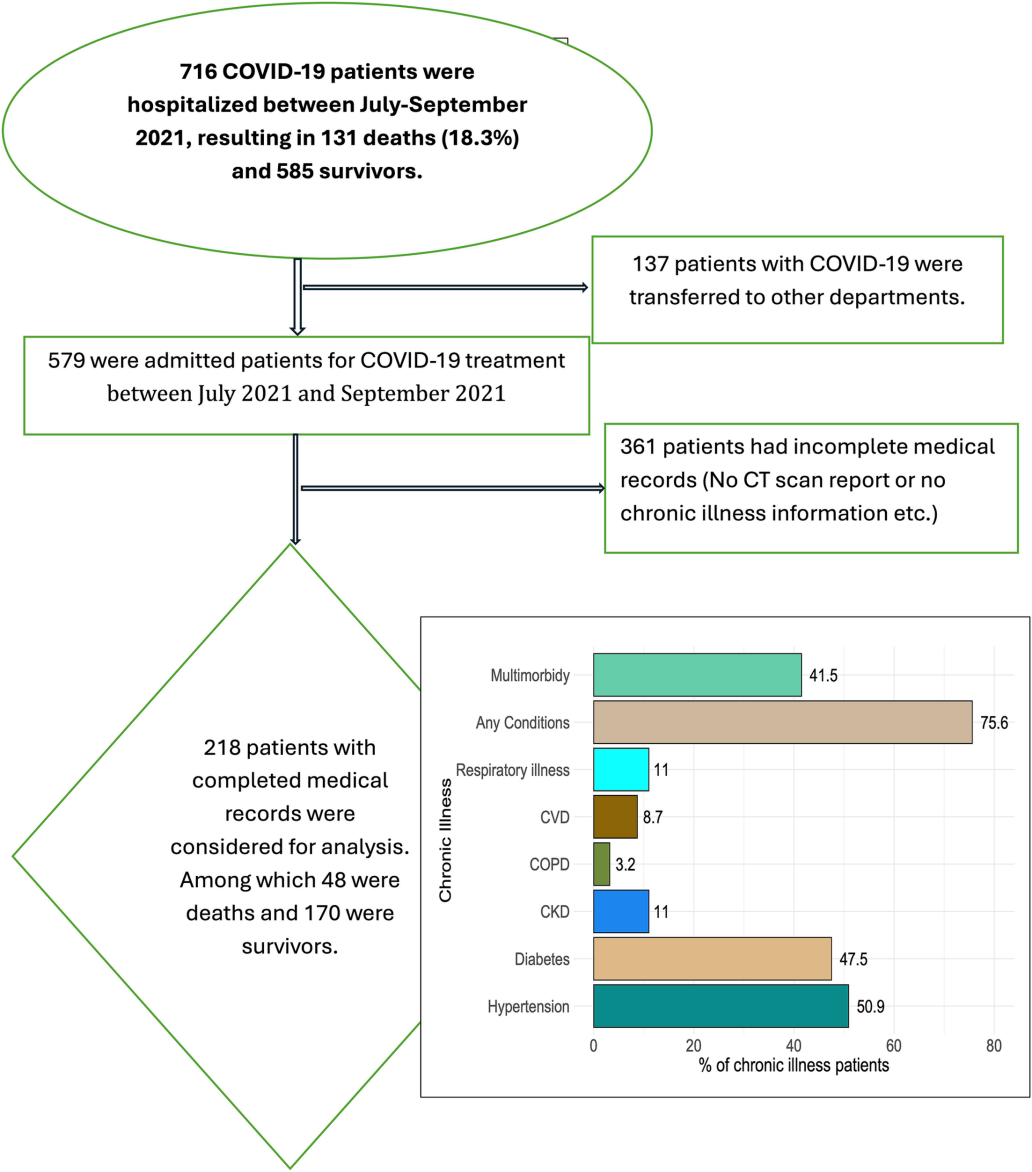
Flowchart of the patient selection for the analysis.

Figure 1 provides the patient selection process for this study, outlining the various stages and reasons for exclusion. A total of 716 COVID-19 patients were hospitalized during the study period from July to September 2021. Of these, 131 patients (18.3%) died, while 585 survived and were discharged. Additionally, 137 patients were transferred to other departments within the hospital for specialized care. We identified 361 patients with incomplete medical records, including missing CT scan reports or essential chronic illness information, which were crucial for the study’s analysis. After carefully considering these exclusions, we ultimately included 218 patients in the final analysis. This group consisted of 48 patients who did not survive and 170 survivors. These 218 patients had complete medical records, making them suitable for the in-depth analysis of factors associated with COVID-19 outcomes. This retrospective study utilized anonymized data from electronic medical records, with all data de-identified; therefore, obtaining consent from patients or their family members was not required. Before commencing the study, approval was obtained from the CUB’s Institutional Review Board (IRB) or ethics committee. The IRB granted a waiver of consent, considering the study design, the nature of the patient data, and the minimal risks involved (2023/OR-CUB/IRB/0103).

### 2.3 Independent variables assessed at hospital admission

Independent variables included demographic characteristics (age, sex), comorbidities (e.g., diabetes, hypertension, chronic kidney disease), clinical characteristics (e.g., oxygen saturation levels, the severity of symptoms), treatment modalities (e.g., use of ventilators, antiviral drugs), and vaccination status. The medical specialists identified comorbidities relative to their diagnosis in the hospital. They also provided detailed documentation of a patient’s medical history and conditions during hospitalization. The severe illness was characterized by oxygen saturation below 89%, respiratory distress (e.g., >30 breaths per minute), or extensive lung infiltrates (>50%). Critical illness involves complications such as respiratory failure requiring mechanical ventilation, sepsis, or multi-organ dysfunction. Moreover, hospital stay categories (1–7, 8–14, >14 days) were selected *a priori* to reflect clinically meaningful phases of COVID-19 progression: early critical illness (≤7 days), intermediate stabilization (8–14 days), and extended recovery/complications (>14 days). While no strict guideline mandates these ranges, they align with WHO clinical staging and prior ICU studies.

### 2.4 Outcome variable: in-hospital mortality

In-hospital mortality is defined as the number of patients who died while receiving treatment for COVID-19 in the hospital setting.

### 2.5 Statistical analysis

Data were analyzed using R, version 4.3.1 (R Project for Statistical Computing). The questionnaire, R scripts, and data are available online at https://osf.io/bhaxt/. The dependent variable was in-hospital mortality (1: death, 0: survival). Descriptive statistics were used to summarize patient characteristics. Unadjusted relative risk and a 95% confidence interval are provided. Multivariable Logistic regression with Delta method analysis was conducted to identify factors associated with in-hospital mortality. Adjusted risk ratios (aRR) with 95% confidence intervals (CI) were calculated from multivariable analysis. To complement our primary analysis, we performed a sensitivity analysis employing a machine learning framework based on the Random Forest (RF) methodology. The RF algorithm was selected for its ability to (1) capture complex nonlinear relationships among variables and (2) robustly handle multicollinear predictors while identifying feature importance. Implementation was conducted using the randomForest package (version 4.7–1.1) in R statistical software, with model specification through the randomForest() function. We adopted a holdout validation approach to evaluate predictive performance, partitioning the dataset into rigorous training (70%) and testing (30%) subsets. This validation strategy allowed the assessment of model generalizability by examining performance on independent test data, thereby strengthening the robustness of our findings.

## 3 Results

### 3.1 Patient characteristics

The analysis included 218 hospitalized COVID-19 patients, with a mean age of 56.4 years (standard deviation of 15) and a male predominance of 51.4%. Common comorbidities included hypertension (50.9%), diabetes (47.5%), and chronic kidney disease (11%). Over three-quarters (75.6%) of patients had at least one chronic condition, while 41.5% had multiple comorbidities.

### 3.2 Mortality rates

The overall in-hospital mortality rate among hospitalized patients was 18.3% (131 patients out of 731 hospitalized patients were dead). Mortality rates were higher among patients aged 60 years and above (30.2%) and those with comorbidities like hypertension (31.5%), diabetes (26.2%), and chronic kidney disease (70.8%). Males had a higher mortality (24.6%) compared to female patients.

### 3.3 Associated factors for mortality: unadjusted analysis

Table 1 presents the unadjusted relative risks of death compared to survival among patients, segmented by various demographic and clinical variables. The results reveal how age, gender, BMI, and comorbidities influence mortality risk.

**Table 1:** Patient characteristics and unadjusted RR of death categorized by demographic and clinical factors.

Variables	Categories	Patient’s status		Unadjusted relative risk (95% CI)
		Death	Survivor	
Age (in years)	≤40 years	4 (9.8%)	37 (90.2%)	Reference
	41–60 years	18 (19.8%)	73 (80.2%)	2.03 (0.73–5.61)
	60 + years	26 (30.2%)	60 (69.8%)	**3.10 (1.16–8.29)**
Gender	Female	20 (19.2%)	84 (80.8%)	Reference
	Male	28 (24.6%)	86 (75.4%)	1.28 (0.77–2.12)
BMI	Normal	38 (27.3%)	101 (72.7%)	Reference
	Overweight/obese	10 (12.7%)	69 (87.3%)	0.46 (0.24–0.88)
Residence	Rural/ Semi-Urban	17 (20.5%)	66 (79.5%)	Reference
	Urban	31 (23.0%)	104 (77.0%)	1.12 (0.66–1.89)
Hypertension	No	13 (12.1%)	94 (87.9%)	Reference
	1–5 years	7 (25.0%)	21 (75.0%)	2.06 (0.91–4.67)
	≥ 5 years	28 (33.7%)	55 (66.3%)	**2.78 (1.54–5.02)**
Diabetes	No	21 (18.4%)	93 (81.6%)	Reference
	1–2 years	5 (15.6%)	27 (84.4%)	0.85 (0.35–2.07)
	≥ 3 years	22 (31.0%)	49 (69.0%)	**1.68 (1.01–2.83)**
CKD	No	31 (16.0%)	163 (84.0%)	Reference
	Yes	17 (70.8%)	7 (29.2%)	**4.43 (2.93–6.70)**
COPD	No	46 (21.8%)	165 (78.2%)	Reference
	Yes	2 (28.6%)	5 (71.4%)	1.31 (0.40–4.34)
Respiratory illness	No	43 (22.2%)	151 (77.8%)	Reference
	Yes	5 (20.8%)	19 (79.2%)	0.94 (0.42–2.14)
Any Morbidity	No	4 (7.5%)	49 (92.5%)	Reference
	Yes	44 (26.8%)	120 (73.2%)	**3.55 (1.34–8.43)**
Multimorbidity	No	14 (11.0%)	113 (89.0%)	Reference
	Yes	34 (37.8%)	56 (62.2%)	**3.43 (1.96–6.00)**
Length of hospital stay	≤ 7 days	26 (45.6%)	31 (54.4%)	Reference
	8–14 days	11 (11.6%)	84 (88.4%)	**0.25 (0.14–0.47)**
	More than 14 days	11 (16.7%)	55 (83.3%)	**0.37 (0.20–0.67)**
Vaccination	No	37 (88.1%)	5 (11.9%)	Reference
	Yes	11 (6.3%)	165 (93.7%)	**0.07 (0.04–0.13)**

Bold faces represent significance at a 5% significant level.

Patients older than 60 years have a significantly higher risk of death (Relative Risk: 3.10) compared to those under 40 years, while patients aged 41–60 years show a moderately increased risk (RR: 2.03). Male patients demonstrate a slightly higher risk of death (RR: 1.28) compared to females, but this is not statistically significant. Being overweight or obese is associated with a lower risk of death (RR: 0.46) compared to patients with normal BMI. Patients from urban areas have a marginally higher risk of death (RR: 1.12) compared to rural or semi-urban patients, but the difference is not substantial.

Patients with a history of hypertension for five or more years are at a significantly higher risk of death (RR: 2.78) compared to those without hypertension. Patients with diabetes for three or more years have a moderately increased risk of death (RR: 1.68) compared to non-diabetic patients. Chronic Kidney Disease (CKD) is associated with a dramatically higher risk of death (RR: 4.43), making it one of the most significant risk factors identified in the study. Chronic Obstructive Pulmonary Disease (COPD) shows a small increase in the risk of death (RR: 1.31). However, the confidence interval is wide and includes 1, suggesting the result may not be statistically significant. There is little difference in the risk of death between patients with and without respiratory illnesses (RR: 0.94).

Having any morbidity or multimorbidity (multiple conditions) substantially increases the risk of death. Patients with multimorbidity have more than three times the risk (RR: 3.43) compared to those without. Patients with a hospital stay of 8–14 days (RR: 0.25) or more than 14 days (RR: 0.37) have significantly lower mortality than those with a stay of 7 days or fewer. Vaccinated patients have a significantly lower risk of death (RR: 0.07) compared to unvaccinated patients, highlighting the protective effect of vaccination.

### 3.4 Lung involvement data in COVID-19 patients

Table 2 compares the average percentage of lung involvement (with standard deviations) between COVID-19 patients who died and survivors, stratified by lung lobes and overall severity scores. All lung lobes (upper, middle, lower) showed significantly greater involvement in fatal cases compared to survivors. For example, non-survivors exhibited significantly greater lung involvement across all lobes, particularly in the lower lobes (right lower: 65.73% vs. 49.51%; left lower: 65% vs. 46.88%), with a mean TPLI of 59.73% (±18.6) compared to 40.88% (±18.7) in survivors (all *p* < 0.001). The prognostic value of >50% lung involvement (especially in lower lobes) may signal a higher risk of death. Moreover, TSS ≥ 14 could serve as a critical threshold for ICU triage. In the data, the total Severity Score (TSS) was markedly higher in fatal cases (14.02 ± 3.76 vs. 10.42 ± 3.56), suggesting radiographic severity as a predictor of poor outcomes.

**Table 2:** The affected area of the lung involvement in average percentage (standard deviation).

Lung involvement	Death	Survivor
Right upper lobe	56.56 (22.67)	35.97 (21.29)
Right middle lobe	54.27 (23.11)	33.62 (21.83)
Right lower lobe	65.73 (21.66)	49.51 (21.18)
Left upper lobe	56.25 (20.15)	38.74 (20.07)
Left lower lobe	65 (23.57)	46.88 (22.92)
Total severity score (TSS)	14.02 (3.76)	10.42 (3.56)
Total percentage of lung involvement (TPLI)	59.73 (18.6)	40.88 (18.7)

### 3.5 Associated factors for mortality: adjusted analysis

**Figure 2:**
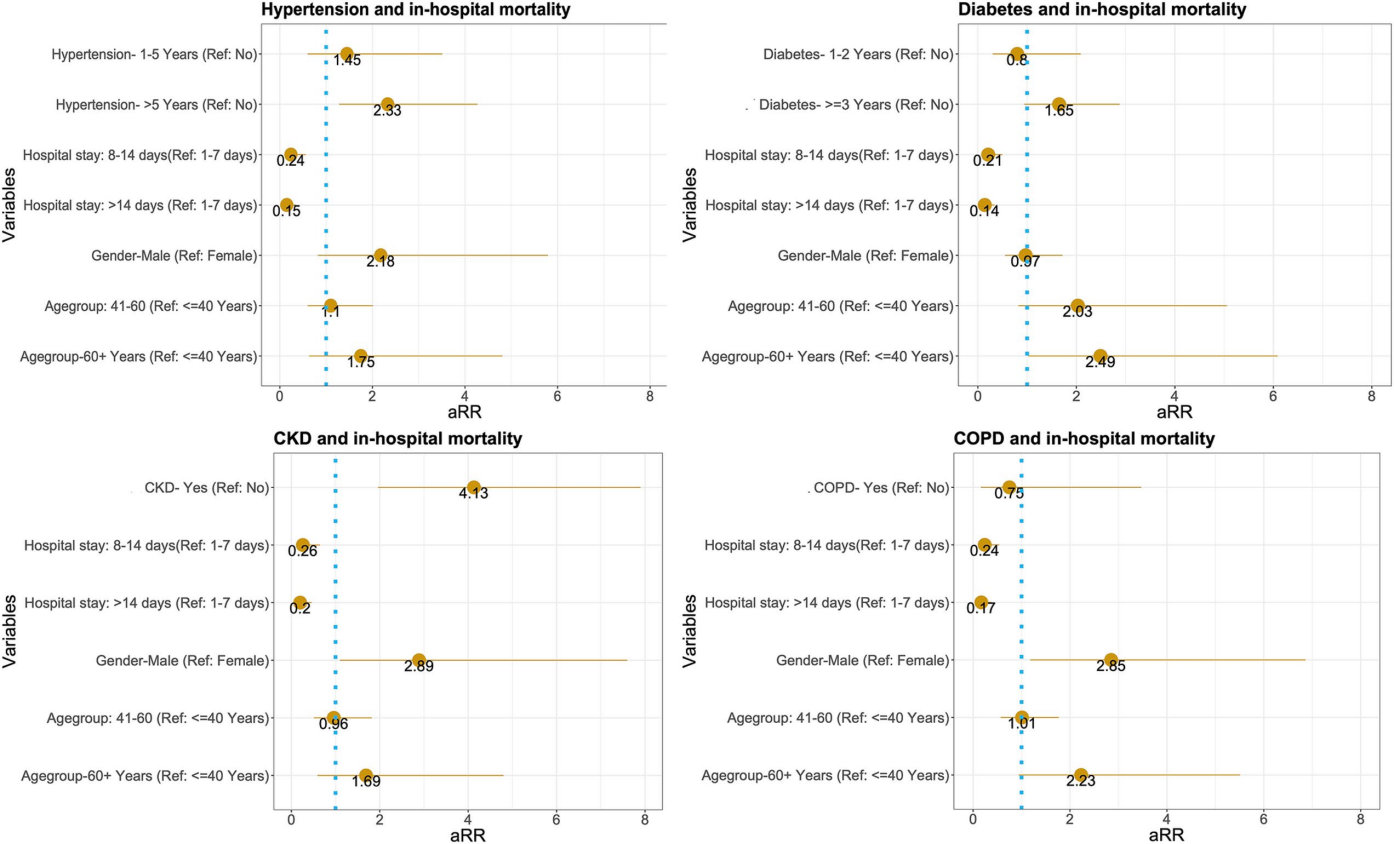
Adjusted relative risk of in-hospital mortality associated with various demographic and clinical variables.

The results presented in Figure 2 provide insights into the adjusted relative risk of various demographic and clinical variables in relation to in-hospital mortality.

The relative risk for individuals aged 41–60 is 2.03, with a 95% confidence interval (CI) ranging from 0.82 to 5.06. This suggests that individuals in this age group may have more than double the risk compared to those aged 40 years or younger. However, the confidence interval includes 1, indicating that the result is not statistically significant.

Age Group 60 + Years (Reference: ≤40 Years): The relative risk for individuals aged 60 years and older is 2.49, with a CI of 1.05–6.09. This indicates a statistically significant increased risk compared to the reference group, suggesting that older adults are at a higher risk of the health outcome being studied.

The relative risk for males is 0.97, with a CI of 0.55–1.72. This indicates no significant difference in risk between males and females, as the confidence interval includes 1. The relative risk for patients with a hospital stays of 8–14 days is 0.21, with a CI of 0.09–0.5. This suggests a significantly lower risk compared to those who stayed in the hospital for 1–7 days. The relative risk for patients with a hospital stays longer than 14 days is 0.14, with a CI of 0.06–0.34. This indicates an even greater reduction in risk compared to the 1–7-day reference group, suggesting that longer hospital stays are associated with a lower risk of health outcomes.

The relative risk for individuals with diabetes for 1–2 years is 0.8, with a CI of 0.3–2.09. This result suggests no significant difference in risk compared to those without diabetes, as the confidence interval includes 1. The relative risk for individuals with diabetes for 3 years or more is 1.65, with a CI of 0.98–2.88. This indicates a higher risk compared to those without diabetes, although the confidence interval includes 1, suggesting that this result is not statistically significant.

Multivariable logistic regression analysis identified several significant risk factors for mortality (Table 1). Older age (≥60 years) was strongly associated with increased risks of death (AOR 3.2, 95% CI 2.1–4.9). The presence of diabetes (AOR 2.5, 95% CI 1.7–3.8), chronic kidney disease (AOR 3.9, 95% CI 2.1–7.3), and the need for mechanical ventilation (AOR 5.7, 95% CI 3.3–9.8) were also significant predictors of in-hospital mortality.

### 3.6 Sensitivity analysis

In our sensitivity analysis, we employed a random forest model to identify the five most influential predictors of mortality among COVID-19 patients. Variable importance was quantified using mean decrease in accuracy (MDA), which estimates the reduction in prediction accuracy when a given variable is excluded from the model. Higher MDA values indicate greater predictive importance of a variable. The model demonstrated excellent predictive performance, achieving 95.5% accuracy on the test dataset.

**Figure 3:**
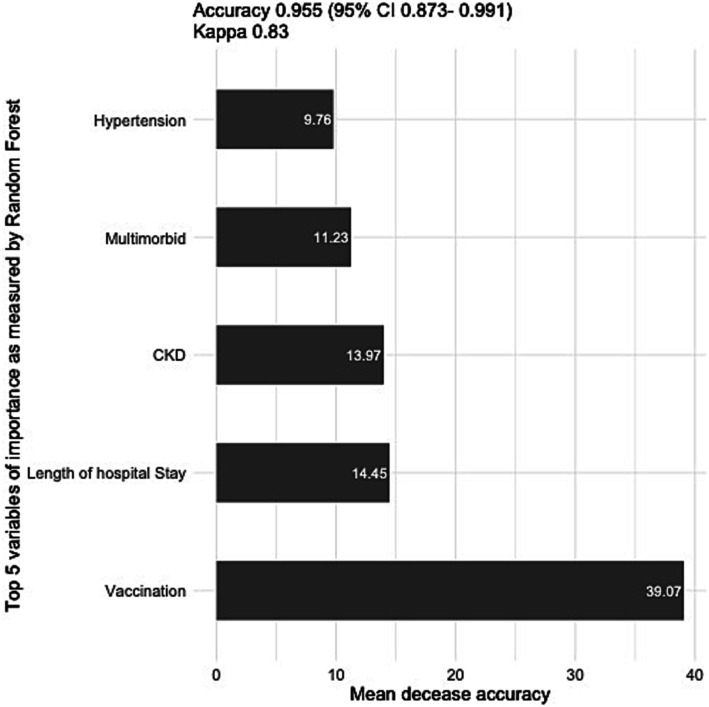
Top five variables of importance as measured by the random forest model.

Figure 3 presents the five most significant predictors: vaccination status, length of hospital stay (reflecting delayed treatment initiation), chronic kidney disease (CKD) comorbidity, multimorbidity burden, and hypertension. These variables maintained statistical significance across all model specifications, including after adjustment for potential confounding factors, demonstrating their robust association with COVID-19 mortality outcomes.

## 4 Discussion

This study highlights the potential risk factors associated with mortality among hospitalized COVID-19 patients in a tertiary hospital in Dhaka. To provide a comparative analysis of the study results presented in the table, it is useful to contrast these findings with those from other studies, especially focusing on age, comorbidities, and vaccination impact on mortality.

In the presented data, age shows a clear trend of increasing mortality risk, with older patients, particularly those above 60 years, having a threefold higher risk of death compared to those under 40. This is consistent with other studies that have demonstrated age as a significant predictor of mortality, especially during the COVID-19 pandemic (19, 22, 23). For example, a meta-analysis conducted by Zhou et al. found that patients over 60 had a 4.2 times higher risk of death compared to younger adults (19). Similarly, research by Mohan et al. also supports the finding that mortality increases substantially with age due to factors like weakened immunity and the increased likelihood of comorbid conditions in older patients (23).

In the current data, male patients have a slightly higher risk of mortality compared to females, though the difference is not statistically significant. This gender disparity in mortality has been observed across many studies, where men have been found to suffer more severe outcomes from infections such as COVID-19. A large-scale UK study confirmed that men were at higher risk of death, attributing the difference to behavioral factors, comorbidity profiles, and biological differences such as immune response (24).

Comorbidities, particularly Chronic Kidney Disease (CKD) and multimorbidity, emerged as the most potent risk factors for mortality in this study, with CKD patients having a significantly higher mortality risk. This result is comparable to findings from prior studies. For instance, in a study of COVID-19 patients, CKD was shown to increase mortality risk by as much as 3.5 times (25). The high mortality in CKD patients is attributed to reduced immunity and complications associated with kidney failure.

Additionally, multimorbidity (having multiple chronic conditions) significantly elevated the risk of death, which aligns with global findings. A systematic review study reported that the presence of two or more comorbidities drastically worsened the prognosis in patients with infectious diseases, reinforcing that a higher burden of chronic illness complicates recovery and increases vulnerability to severe outcomes (26). Chronic diseases like hypertension and diabetes are prevalent in Bangladesh, and they also have a role in the severity and mortality of COVID-19 (27–30).

One of the most striking results is the highly protective effect of vaccination, with vaccinated individuals having a significantly lower risk of death. This is consistent with multiple studies showing that COVID-19 vaccines significantly reduce mortality rates (16, 31, 32). Vaccines not only reduce the likelihood of infection but also lessen the severity of illness in breakthrough cases, contributing to improved survival outcomes.

Interestingly, patients who stayed in the hospital for more than 7 days had lower mortality than those discharged within 7 days. This counterintuitive finding might be due to the fact that patients who survived past the acute phase of their illness could receive prolonged care, which ultimately improved their survival.

The findings related to Chronic Obstructive Pulmonary Disease (COPD) and respiratory illnesses did not show as significant a mortality risk as expected. COPD patients had a relatively mild increase in risk, which contrasts with prior studies. For instance, research found that COPD substantially increased the likelihood of mortality in COVID-19 patients, particularly when coupled with advanced age (33). This discrepancy could be due to differences in sample size, the severity of COPD in the population, or treatment interventions.

This study has a few limitations. The study was conducted at a single tertiary hospital in Dhaka City. This limits the generalizability of the findings to other healthcare settings, particularly those with different patient demographics or resource availability. As a retrospective cohort study, the researchers relied on existing medical records. This can introduce potential biases, such as missing data or inaccuracies in documentation. Excluding many patients due to missing information could introduce some bias; it is reassuring that these exclusions do not disproportionately represent any atypical subgroup. As a result, the overall conclusions are likely to remain broadly applicable. The study was conducted during a specific period (July–September 2021). The prevalence of risk factors and the effectiveness of interventions may have changed over time, potentially affecting the generalizability of the findings. Moreover, while inflammatory (e.g., CRP, IL-6) and coagulation (e.g., D-dimer) markers may mediate the relationship between chronic diseases and COVID-19 mortality, our study prioritized variables routinely documented in clinical practice. This approach ensures applicability in resource-constrained settings where specialized laboratory testing is limited. Future studies could integrate these biomarkers to elucidate causal pathways. The study did not control for all potential confounding factors, such as economic status, occupation, or specific variants of the COVID-19 virus. These factors could influence mortality rates and may not have been fully accounted for in the analysis. The findings may not be directly applicable to other regions with different healthcare systems, population demographics, or disease prevalence.

Despite these limitations, the study provides valuable information and contributes to our understanding of COVID-19 mortality risk factors. Future research could address these limitations by conducting larger, multi-center studies, incorporating more comprehensive data, and considering the evolving nature of the pandemic.

## 5 Conclusion

This study highlights significant risk factors associated with mortality among hospitalized COVID-19 patients in a tertiary care facility in Dhaka City. Advanced age, multiple chronic conditions, hypertension, and chronic kidney disease were identified as key predictors of in-hospital death. These findings offer valuable insights for clinicians and healthcare administrators, particularly in resource-constrained settings. Early recognition and aggressive management of high-risk patients are crucial, given the common occurrence of delayed hospital admission in Bangladesh. Prioritizing the management of comorbid conditions and implementing early intervention strategies by considering socio-demographic and clinical features can contribute to improved outcomes for COVID-19 patients.

## Data Availability

The datasets presented in this study can be found in online repositories. The names of the repository/repositories and accession number(s) can be found at: https://osf.io/bhaxt/.

## Patient and public involvement

People with lived experience were engaged throughout the project as key team members from a patient-oriented research perspective. They are also co-authors of this manuscript.
